# Supplementary material for: Thyroid carcinoma in children, adolescents, and young adults in Brazil: A report from 11 population-based cancer registries
Source: PLoS One. 2020 May 1;15(5):e0232416. doi: 10.1371/journal.pone.0232416 (PMC7194432; doi:10.1371/journal.pone.0232416)
Supplement: S1 Table — (DOCX) [file pone.0232416.s001.docx]

Table S1: Numbers of cases and age-specific incidence rates of thyroid carcinomas in five Brazilian Regions, 11 PBCR in cases aged <40 years and diagnosed during the period 2000-2013

| **Gender** | **Local** | **Age-group** | | | | | | | | | | | | | |
| --- | --- | --- | --- | --- | --- | --- | --- | --- | --- | --- | --- | --- | --- | --- | --- |
|  |  | **0-9** | | **10-14** | | **15-19** | | **20-24** | | **25-29** | | **30-34** | | **35-39** | |
|  |  | **N** | **ASR** | **N** | **ASR** | **N** | **ASR** | **N** | **ASR** | **N** | **ASR** | **N** | **ASR** | **N** | **ASR** |
| **Total** | **North** | ****** | **0.39** | ****** | **1.46** | **10** | **3.35** | **28** | **9.22** | **43** | **15.05** | **38** | **15.19** | **53** | **24.8** |
|  | Belém | ** | 0.44 | ** | 1.22 | 9 | 3.36 | 22 | 8.14 | 37 | 14.54 | 37 | 16.56 | 46 | 23.87 |
|  | Palmas | 0 | 0.00 | ** | 3.64 | ** | 3.26 | 6 | 17.95 | 6 | 19.18 | ** | 3.73 | 7 | 33.27 |
|  | **Northeast** | ****** | **0.34** | **8** | **2.40** | **57** | **15.73** | **93** | **24.98** | **157** | **44.75** | **239** | **74.76** | **267** | **92.35** |
|  | Aracaju | 0 | 0.00 | ** | 7.58 | 20 | 27.42 | 42 | 54.84 | 95 | 133.35 | 150 | 237.69 | 171 | 311.33 |
|  | João Pessoa | 0 | 0.00 | ** | 3.53 | 9 | 9.65 | 20 | 21.06 | 19 | 21.58 | 41 | 50.79 | 28 | 39.14 |
|  | Recife | ** | 0.62 | 0 | 0.00 | 28 | 14.28 | 31 | 15.45 | 43 | 22.45 | 48 | 27.29 | 68 | 41.80 |
|  | **Midwest** | **0** | **0.00** | ****** | **3.50** | **42** | **25.29** | **81** | **45.06** | **131** | **77.04** | **164** | **106.8** | **168** | **124.56** |
|  | Goiânia | 0 | 0.00 | ** | 3.50 | 42 | 25.29 | 81 | 45.06 | 131 | 77.04 | 164 | 106.80 | 168 | 124.56 |
|  | **Southeast** | **20** | **0.67** | **83** | **5.18** | **349** | **20.35** | **897** | **48.16** | **1 768** | **95.85** | **2 806** | **163.27** | **3 114** | **199.59** |
|  | Barretos | ** | 1.18 | ** | 2.05 | 8 | 15.65 | 15 | 29.91 | 16 | 33.74 | 30 | 67.40 | 40 | 97.71 |
|  | Belo Horizonte | 0 | 0.00 | ** | 1.15 | 25 | 8.56 | 41 | 12.77 | 87 | 28.30 | 107 | 37.60 | 114 | 44.45 |
|  | Jahu | 0 | 0.00 | 0 | 0.00 | ** | 26.46 | ** | 19.40 | ** | 33.10 | 11 | 77.49 | 13 | 96.45 |
|  | São Paulo | 19 | 0.79 | 79 | 6.17 | 312 | 22.95 | 838 | 56.78 | 1 660 | 112.58 | 2 658 | 193.26 | 2 947 | 236.14 |
|  | **South** | **0** | **0.00** | **15** | **6.11** | **25** | **11.58** | **57** | **24.65** | **90** | **39.72** | **138** | **64.75** | **130** | **66.56** |
|  | Curitiba | 0 | 0.00 | 15 | 6.11 | 25 | 11.58 | 57 | 24.65 | 90 | 39.72 | 138 | 64.75 | 130 | 66.56 |
|  | **Brazilian pool** | **24** | **0.51** | **112** | **4.39** | **483** | **17.51** | **1 156** | **39.20** | **2 189** | **76.70** | **3 385** | **127.48** | **3 732** | **155.94** |
| **Females** | **North** | ****** | **0.40** | ****** | **2.91** | **9** | **5.81** | **24** | **15.07** | **36** | **23.91** | **34** | **25.71** | **43** | **38.23** |
|  | Belém | ** | 0.44 | ** | 2.43 | 8 | 5.77 | 19 | 13.42 | 30 | 22.37 | 33 | 27.88 | 37 | 36.33 |
|  | Palmas | 0 | 0.00 | ** | 7.16 | ** | 6.13 | 5 | 28.34 | 6 | 36.47 | ** | 7.21 | 6 | 56.33 |
|  | **Northeast** | ****** | **0.68** | ****** | **3.02** | **40** | **21.73** | **74** | **38.21** | **135** | **72.56** | **207** | **120.45** | **237** | **150.69** |
|  | Aracaju | 0 | 0.00 | ** | 9.10 | 13 | 34.56 | 33 | 81.53 | 84 | 219.66 | 130 | 379.39 | 151 | 502.16 |
|  | João Pessoa | 0 | 0.00 | ** | 4.72 | ** | 10.51 | 17 | 34.29 | 19 | 40.57 | 35 | 80.88 | 26 | 66.96 |
|  | Recife | ** | 1.26 | 0 | 0.00 | 22 | 22.25 | 24 | 23.17 | 32 | 31.69 | 42 | 44.53 | 60 | 67.89 |
|  | **Midwest** | **0** | **0.00** | ****** | **4.22** | **32** | **38.13** | **72** | **77.64** | **108** | **121.69** | **138** | **170.89** | **137** | **190.79** |
|  | Goiânia | 0 | 0.00 | ** | 4.22 | 32 | 38.13 | 72 | 77.64 | 108 | 121.69 | 138 | 170.89 | 137 | 190.79 |
|  | **Southeast** | **13** | **0.86** | **59** | **7.20** | **290** | **32.53** | **763** | **78.16** | **1 493** | **153.25** | **2 382** | **260.73** | **2 579** | **308.14** |
|  | Barretos | 0 | 0.00 | ** | 4.22 | 6 | 23.89 | 12 | 48.79 | 12 | 50.87 | 27 | 12.94 | 30 | 140.39 |
|  | Belo Horizonte | 0 | 0.00 | ** | 1.54 | 21 | 14.19 | 35 | 21.21 | 78 | 48.83 | 84 | 56.83 | 92 | 67.60 |
|  | Jahu | 0 | 0.00 | 0 | 0.00 | ** | 53.26 | ** | 26.46 | ** | 66.67 | 10 | 140.19 | 11 | 161.42 |
|  | São Paulo | 13 | 1.06 | 56 | 8.50 | 256 | 36.43 | 714 | 91.66 | 1 398 | 178.45 | 2 261 | 307.56 | 2 446 | 363.62 |
|  | **South** | **0** | **0.00** | **8** | **8.25** | **20** | **18.50** | **48** | **41.09** | **76** | **65.57** | **118** | **107.37** | **115** | **111.87** |
|  | Curitiba | 0 | 0.00 | 8 | 8.25 | 20 | 18.50 | 48 | 41.09 | 76 | 65.57 | 118 | 107.37 | 115 | 111.87 |
|  | **Brazilian pool** | **16** | **0.68** | **79** | **6.12** | **391** | **27.49** | **981** | **63.75** | **1 848** | **121.94** | **2 879** | **204.43** | **3 111** | **248.80** |
| **Males** | **North** | ****** | **0.38** | **0** | **0.00** | ****** | **0.70** | ****** | **2.77** | **7** | **5.11** | ****** | **3.39** | **10** | **9.88** |
|  | Belém | ** | 0.43 | 0 | 0.00 | ** | 0.77 | ** | 2.33 | 7 | 5.82 | ** | 3.81 | 9 | 9.91 |
|  | Palmas | 0 | 0.00 | 0 | 0.00 | 0 | 0.00 | ** | 6.34 | 0 | 0.00 | 0 | 0.00 | ** | 9.63 |
|  | **Northeast** | **0** | **0.00** | ****** | **1.79** | **17** | **9.54** | **19** | **10.64** | **22** | **13.35** | **32** | **21.65** | **30** | **22.75** |
|  | Aracaju | 0 | 0.00 | ** | 6.06 | 7 | 19.82 | 9 | 24.92 | 11 | 33.33 | 20 | 69.34 | 20 | 80.47 |
|  | João Pessoa | 0 | 0.00 | ** | 2.34 | ** | 8.76 | ** | 6.61 | 0 | 0.00 | 6 | 16.02 | ** | 6.12 |
|  | Recife | 0 | 0.00 | 0 | 0.00 | 6 | 6.17 | 7 | 7.21 | 11 | 12.15 | 6 | 7.36 | 8 | 10.77 |
|  | **Midwest** | **0** | **0.00** | ****** | **2.79** | **10** | **12.47** | **9** | **10.37** | **23** | **28.29** | **26** | **35.71** | **31** | **49.15** |
|  | Goiânia | 0 | 0.00 | ** | 2.79 | 10 | 12.47 | 9 | 10.37 | 23 | 28.29 | 26 | 35.71 | 31 | 49.15 |
|  | **Southeast** | **7** | **0.47** | **24** | **3.06** | **59** | **7.14** | **134** | **15.12** | **275** | **31.60** | **424** | **52.67** | **535** | **73.97** |
|  | Barretos | ** | 2.29 | 0 | 0.00 | ** | 7.69 | ** | 11.74 | ** | 16.78 | ** | 13.52 | 10 | 47.93 |
|  | Belo Horizonte | 0 | 0.00 | ** | 0.76 | ** | 2.78 | 6 | 3.85 | 9 | 6.09 | 23 | 16.97 | 22 | 18.28 |
|  | Jahu | 0 | 0.00 | 0 | 0.00 | 0 | 0.00 | ** | 12.66 | 0 | 0.00 | ** | 14.16 | ** | 30.01 |
|  | São Paulo | 6 | 0.51 | 23 | 3.70 | 53 | 8.17 | 124 | 17.79 | 262 | 37.91 | 397 | 62.02 | 501 | 87.08 |
|  | **South** | **0** | **0.00** | ****** | **4.02** | ****** | **4.64** | **9** | **7.87** | **14** | **12.65** | **20** | **19.38** | **15** | **16.21** |
|  | Curitiba | 0 | 0.00 | ** | 4.02 | ** | 4.64 | 9 | 7.87 | 14 | 12.65 | 20 | 19.38 | 15 | 16.21 |
|  | **Brazilian pool** | **8** | **0.34** | **33** | **2.62** | **92** | **6.89** | **175** | **12.41** | **341** | **25.03** | **506** | **40.58** | **621** | **55.85** |

* less than 5 cases;

Abbreviations: N: number of cases; ASR: age-specific rate; PBCR population-based cancer registries
